# Effects of Long-Chain Fatty Acyl-CoA Synthetase 1 on Diglyceride Synthesis and Arachidonic Acid Metabolism in Sheep Adipocytes

**DOI:** 10.3390/ijms21062044

**Published:** 2020-03-17

**Authors:** Yang Cao, Sutian Wang, Shunqi Liu, Yanli Wang, Haiguo Jin, Huihai Ma, Xiaotong Luo, Yang Cao, Zhengxing Lian

**Affiliations:** 1 Beijing Key Laboratory for Animal Genetic Improvement, National Engineering Laboratory for Animal Breeding, Key Laboratory of Animal Genetics and Breeding of the Ministry of Agriculture, College of Animal Science and Technology, China Agricultural University, Beijing 100193, China; B20163040277@cau.edu.cn (Y.C.); liusq23@cau.edu.cn (S.L.); 2 Branch of Animal Husbandry, Jilin Academy of Agricultural Science, Gongzhuling 136100, China; wyl940325@163.com (Y.W.); khk1962@126.com (H.J.); mh2pc2013@163.com (H.M.); XiaotongLuo2019@163.com (X.L.); 3 State Key Laboratory of Livestock and Poultry Breeding, Institute of Animal Science, Guangdong Academy of Agricultural Sciences, Guangzhou 510640, China; wstlyt@cau.edu.cn

**Keywords:** ACSL1, RNA-seq, lipid metabolome, arachidonic acid, COX2

## Abstract

Long-chain fatty acyl-CoA synthetase (ACSLs) is an essential enzyme for the synthesis of fatty acyl-CoA. ACSL1 plays a key role in the synthesis of triglycerides, phospholipids, and cholesterol esters. Background: In the current study, triglyceride content did not increase after overexpression of the *ACSL1* gene. Methods: RNA-seq and lipid metabolome profiling were performed to determine why triglyceride levels did not change with *ACSL1* overexpression. Results: Fatty acyl-CoA produced by ACSL1 was determined to be involved in the diglyceride synthesis pathway, and diglyceride content significantly increased when *ACSL1* was overexpressed. Moreover, the arachidonic acid (AA) content in sheep adipocytes significantly increased, and the level of cyclooxygenase 2 (COX2) expression, the downstream metabolic gene, was significantly downregulated. Knocking down the *ACSL1* gene was associated with an increase in *COX2* mRNA expression, as well as an increase in prostaglandin content, which is the downstream metabolite of AA. Conclusions: The overexpression of the ACSL1 gene promotes the production of AA via downregulation of COX2 gene expression.

## 1. Introduction

Long-chain fatty acyl-CoA synthetases (ACSLs) are essential for fatty acid (FA) activation and catalysis. ACSL1 converts long-chain FAs to fatty acyl-CoA. Studies have shown that ACSLs play key roles in the synthesis of triglycerides, phospholipids, and cholesterol esters, as well as in the metabolism of FA [1]. The *ACSL1* gene encodes a synthetase that is involved in lipid metabolism and mainly expressed in adipose tissues and the inner membrane of the liver [2]. ACSL1 is also involved in the synthesis of triglycerides from fatty acyl-CoA, promotes the deposition of FAs [3], activates FA, and enters the β-oxidation pathway [4].

Previous studies have shown that the *ACSL1* gene affects FA composition by adjusting the total fat content of skeletal muscle [5]. In addition, ACSL1 was also found to influence the relative content of different fractions of unsaturated FAs, omega-3 FAs, polyunsaturated FAs, long-chain omega-3 FAs, and docosapentaenoic acid [6]. ACSL1 is a major subtype that promotes triglyceride synthesis in 3T3-L1 adipocytes [2]. In mice, which was specifically overexpressed with the *ACSL1* gene, triglyceride levels increased 12-fold, and choline glycerophospholipids increased 1.5-fold [7]. Carlos et al. reported that *ACSL1* gene expression gradually increases as individuals grow and reaches the maximum level after adulthood [8]. Suzuki et al. found that high-carbohydrate foods and high-fat diets affect the expression of ACSL1 in the liver [9]. Loss of ACSL1 causes changes in the expression of pro-inflammatory chemokines and downregulates the amount of cellular lipids and glucose uptake [10]. ACSL1 promotes differentiation of brown adipocytes, and ACSL1 deficiency in brown adipocytes reduces body weight in mice fed a high-fat diet [11]. FA oxidation levels in white adipocytes decrease, and cold tolerance is low in ACSL1 knockout mice, indicating that ACSL1 plays an important role in the activation of FA oxidation [12]. These studies suggest that the level of ACSL1 in tissues may be related to FA metabolism.

The quality, texture, and taste of meat are related to intramuscular adipocytes [13]. Unsaturated FAs in mutton not only affect taste but are also essential nutrients for humans [14]. In an initial experiment, Du Han sheep (Duper sheep × Small-Tailed Han sheep cross F1 generation) and Small-Tailed Han sheep were studied. First, a comparative analysis of production performance was conducted. The results showed that the mean carcass weight of the crossbred sheep was significantly higher than that of the Small-Tailed Han sheep. Genome-wide methylation sequencing was performed using methylated DNA immunoprecipitation sequencing on two populations of Duhan sheep and Small-Tailed Han sheep and showed that the expression level of ACSL1 was significantly different between the two groups (*p* < 0.05) and that the *ACSL1* gene is regulated by methylation thereby affecting lipid metabolism and meat quality [15]. The current study explored the regulatory mechanism of the *ACSL1* gene on intramuscular fat deposition in sheep adipocytes to provide a theoretical and scientific basis for breeding. In this study, the coding sequence (CDS) region of *ACSL1* was cloned and transfected into sheep preadipocytes. After the cells were allowed to differentiate for eight days, and cellular triglyceride content was measured, the triglyceride content in the treated cells did not significantly differ from the control cells. This result is in contrast to what was previously known about the role of the *ACSL1* gene in lipid metabolism [1,2,7]. To further determine the effect of ACSL1 on sheep adipocytes, a combined transcriptome and metabolome analysis was performed.

## 2. Results

### 2.1. Overexpression of Sheep ACSL1 Gene in Sheep Adipocytes

**Figure 1:**
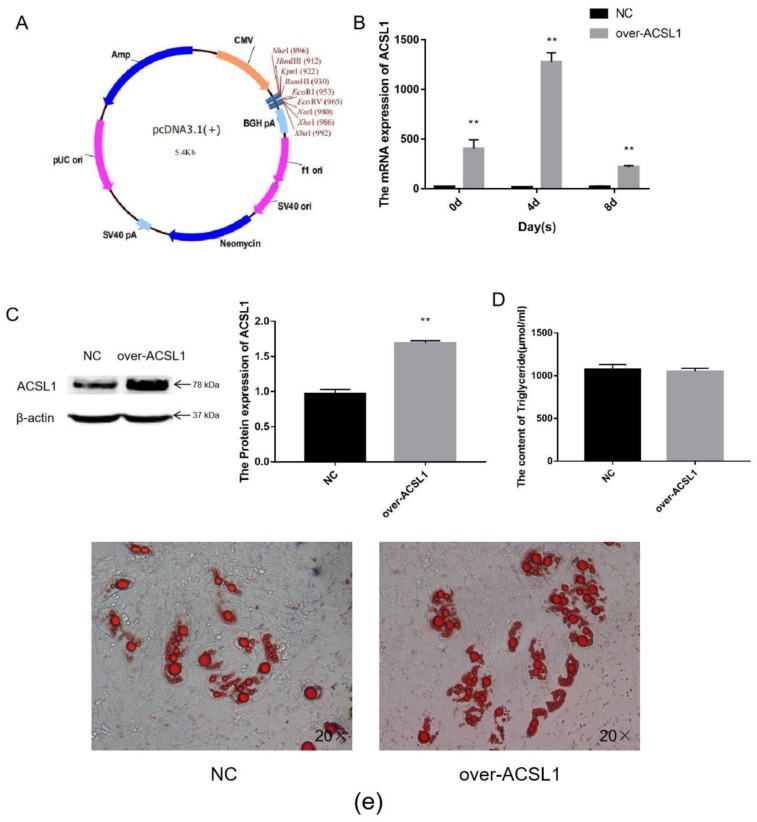
Overexpression of the *ACSL1* gene in sheep preadipocytes: (**a**) the pcDNA3.1(+) vector; (**b**) mRNA expression of the *ACSL1* gene on days 0, 4, and 8 of differentiation (* *p* < 0.05, ** *p* < 0.01); (**c**) the protein expression of the ACSL1 gene on day 8 of differentiation; (**d**) the detection of intracellular triglyceride content; (**e**) oil red staining of adipocytes after induced differentiation on day 8 (Left: control; Right: overexpression ACSL1). NC: Adipocytes transfected with pcDNA3.1(+) vector for control. Over-ACSL1: Adipocytes transfected with pcDNA3.1(+) vector with *ACSL1* CDS.

The CDS region of the *ACSL1* gene was cloned, and the fragment ligated into the pcDNA3.1(+) vector (Figure 1a) via BamHI and EcoRI digestion. The vectors were transfected into sheep preadipocytes. The expression of *ACSL1* mRNA in sheep adipocytes significantly increased during the process of inducing differentiation on days 0, 4, and 8 (Figure 1b), and protein expression also significantly increased on day 8 (Figure 1c). On the 8th day after induction of differentiation, the transfected cells developed into mature adipocytes. Large lipid droplets were observed, which is consistent with the form of fat in the body (Figure 1e). These findings demonstrate that ACSL1-overexpressing preadipocytes were successfully generated. However, the triglyceride content comparing the overexpressing adipocytes with control adipocytes did not significantly differ (Figure 1d).

### 2.2. RNA-seq and Lipid Metabolome Analyses

**Figure 2:**
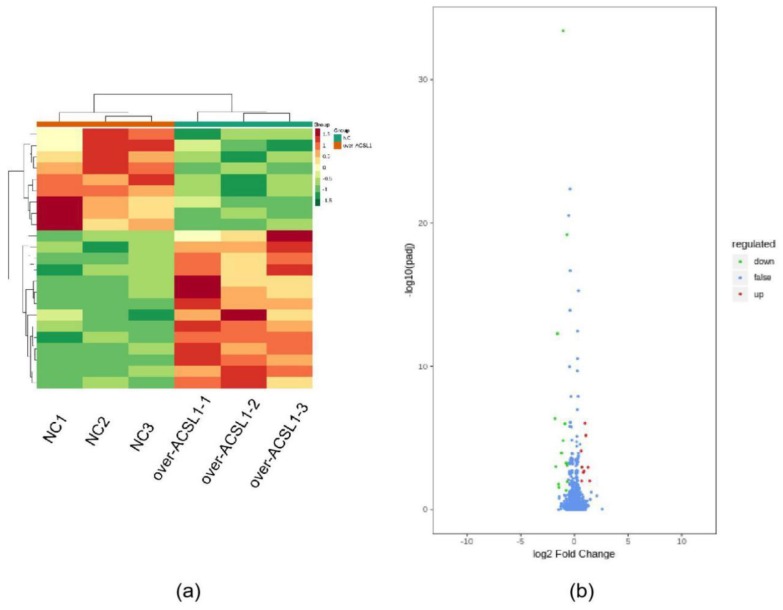

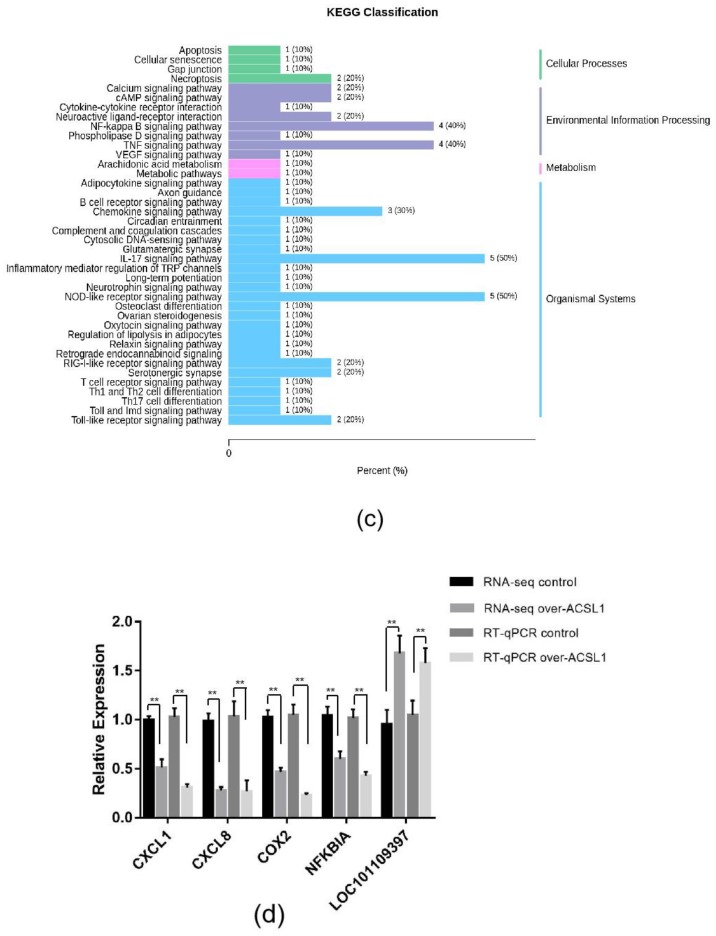
Transcriptome sequencing: (**a**) heat map; (**b**) volcano plots; (**c**) KEGG analysis; (**d**) RNA-seq data and qRT-PCR of sheep adipocytes (* *p* < 0.05). NC: Adipocytes transfected with pcDNA3.1(+) vector as control. Over-ACSL1: Adipocytes transfected with pcDNA3.1(+) vector with *ACSL1* CDS.

**Figure 3:**
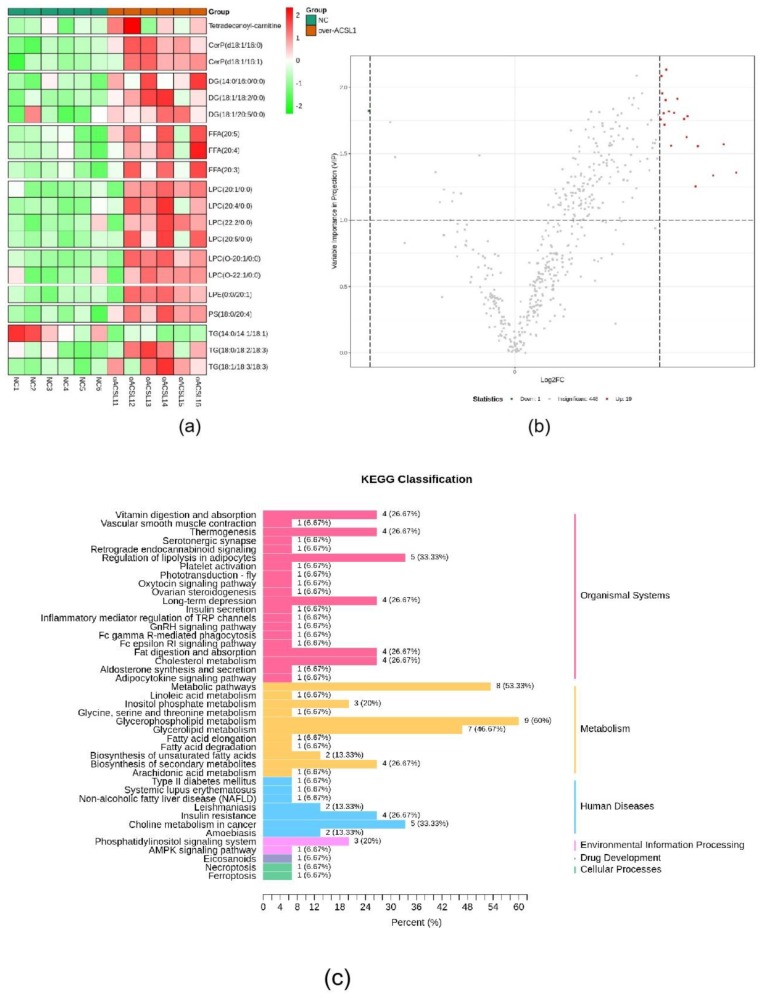

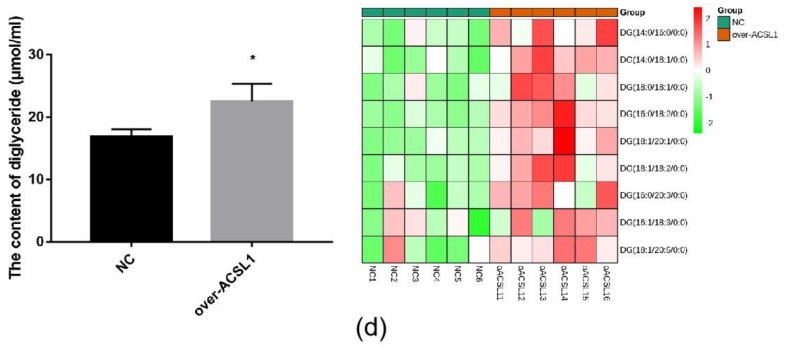
Lipid metabolome sequencing: (**a**) heat map; (**b**) volcano plots; (**c**) KEGG analysis; (**d**) the detection of intracellular diglyceride content (* *p* < 0.05). NC: Adipocytes transfected with pcDNA3.1(+) vector as control. Over-ACSL1: Adipocytes transfected with pcDNA3.1(+) vector with *ACSL1* CDS.

RNA-seq and lipid metabolome profiling were used to sequence sheep adipocytes overexpressing the *ACSL1* gene. Twenty-three differentially expressed genes (DEGs) were screened using RNA-seq, of which 14 DEGs were upregulated, and 9 genes were downregulated (Figure 2a,b). The DEGs were mainly related to the IL-17 signaling pathway, arachidonic acid (AA) metabolism, and the nucleotide-binding oligomerization domain-like receptors signaling pathway (Figure 2c). RT-PCR verified that the differential gene expression results coincided with the sequencing findings. Growth-regulated alpha protein precursor (CXCL1), interleukin-8 precursor (CXCL8), NF-kappa-B inhibitor alpha (NFKBIA), and cyclooxygenase 2 (COX2) were all significantly downregulated, and histone H1.3 (LOC101109397) was significantly upregulated (Figure 2d) in the overexpressing adipocytes compared to the controls. A total of 469 metabolites were detected in the metabolome. There were 20 differential metabolites, of which 19 were upregulated and 1 was downregulated (Figure 3a,b). The differential metabolites were mainly associated with the classification of eicosanoids, free FAs, lysophosphatidylcholine, acylcarnitine, ceramide, diglyceride, phosphatidylserine, and triglycerides (Figure 3c, Table 1). FFA(20:3) are activated to promote the synthesis of acly-CoA, generating acyl-CoA into the diglyceride, triglyceride synthesis pathway. All the indicators of diglycerides were increased in the metabolome sequencing, but the difference was not significant. Furthermore, total diglyceride content of the differentiated adipocytes was measured. The diglyceride content in the overexpressing ACSL1 adipocytes was higher than in the controls (Figure 3d). This result is consistent with the lipid metabolome sequencing findings.

### 2.3. Effect of the ACSL1 Gene on the AA Metabolic Pathway

**Figure 4:**
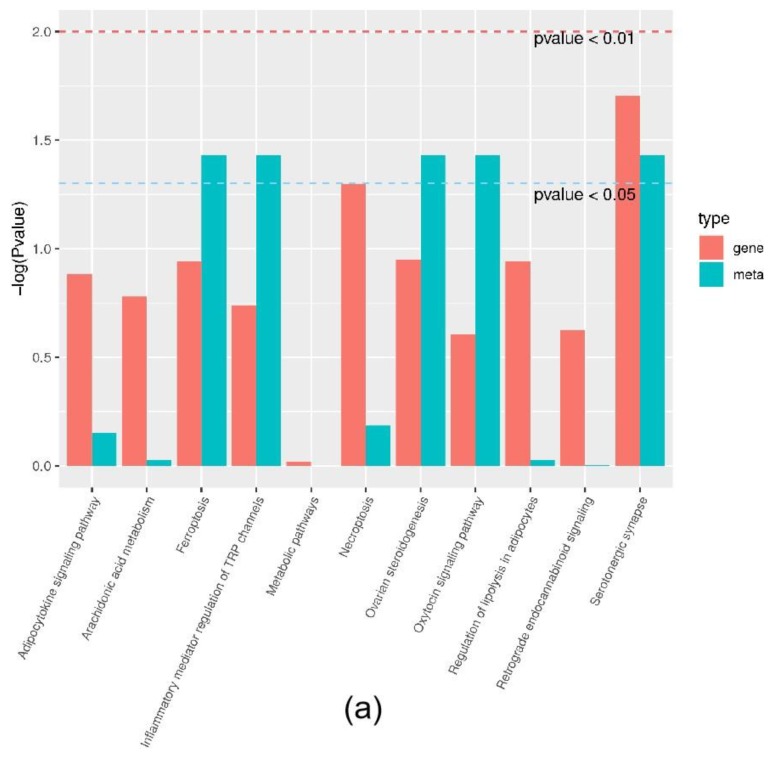

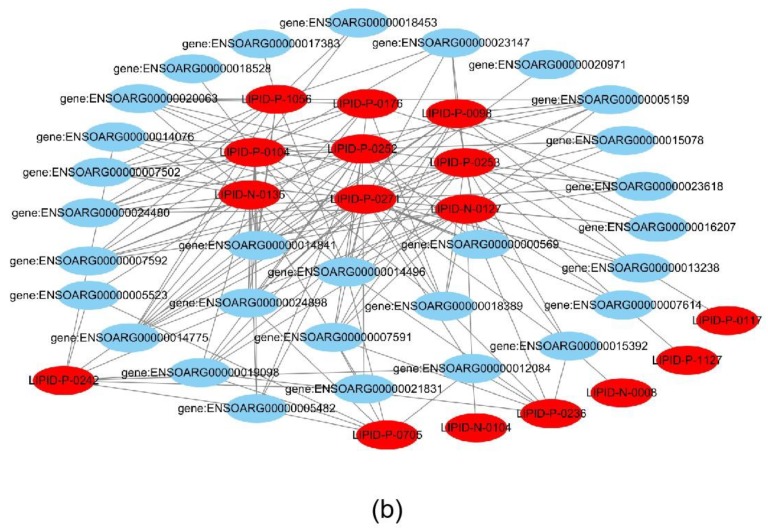
Joint analysis results: (**a**) the classification histogram of pathways in which differentially expressed genes and differential metabolites were enriched in KEGG analysis; (**b**) correlation network diagram of differentially expressed genes and differential metabolites. Red: differential metabolites; Blue: differentially expressed genes.

**Figure 5:**
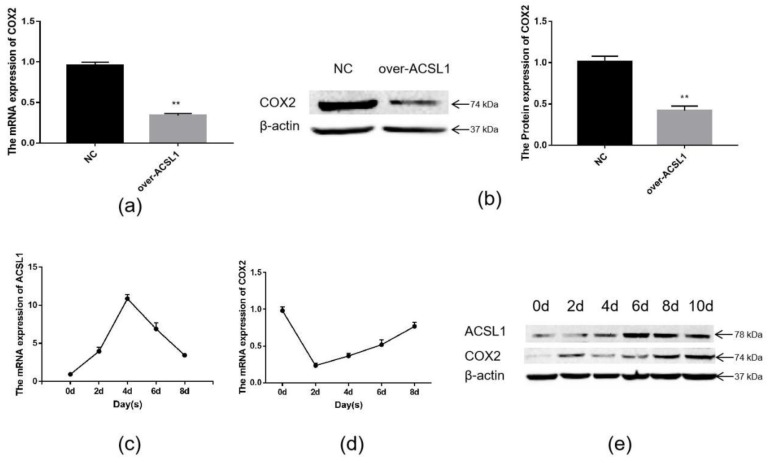
The expression of ACSL1 and COX2: (**a**) (**b**) the mRNA and protein expression of COX2 in adipocytes with overexpression of the *ACSL1* gene on day 8 after differentiation; (**c**) the mRNA expression of the *ACSL1* in different stages of differentiation; (**d**) the mRNA expression of the *COX2* gene in different stages of differentiation; (**e**) the protein expression of the ACSL1 and COX2 genes in different stages of differentiation; (* *p* < 0.05, ** *p* < 0.01). NC: Adipocytes transfected with the pcDNA3.1(+) vector as control. Over-ACSL1: Adipocytes transfected with the pcDNA3.1(+) vector with *ACSL1* CDS.

Joint analysis revealed a correlation between DEGs and differential metabolites. These partly clustered the same signaling pathway (Figure 4a), and correlations between DEGs and differential metabolites were mapped (Figure 4b). Differential metabolites of AA and the DEG *COX2* were found in the AA metabolic pathway. AA is an omega-6 polyunsaturated FA, which is a relative of saturated peanut acid in peanut oil. COX is an important enzyme in the metabolism of AA, and the metabolite of COX is prostaglandin (PG). In the lipid metabolome assay, the AA content [FFA (20:4) in Table 1] in the overexpression group was significantly higher than that in the control group. The mRNA expression of the *COX2* gene, which is an important metabolism-related gene downstream of AA, was significantly lower in the overexpression group compared to the control group as shown by RNA-seq. Both RT-PCR and Western blotting verified this result (Figure 5a,b). Similarly, the expression trends of the ACSL1 and COX2 genes were found to be opposite during the differentiation of sheep adipocytes (Figure 5c–e).

**Figure 6:**
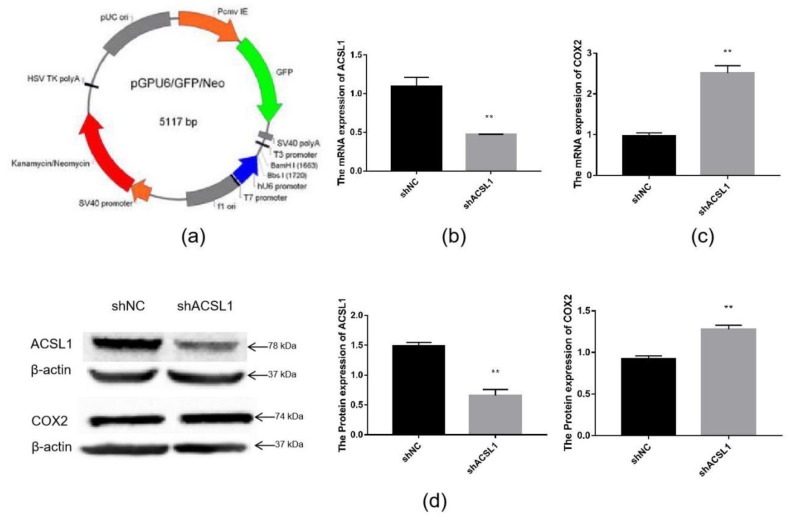
Knockdown of the ACSL1 gene in sheep preadipocytes: (**a**) the pGPU6 vector; (**b**) *ACSL1* mRNA expression on day 8 of differentiation (** *p* < 0.01); (**c**) *COX2* mRNA expression on day 8 of differentiation (** *p* < 0.01); (**d**) ACSL1 and COX2 protein expression on day 8 of differentiation (** *p* < 0.01). shNC: Adipocytes transfected with shNC as control. shACSL1: Adipocytes transfected with shRNA-1928 of the *ACSL1* gene.

To further verify the effect of the *ACSL1* gene on AA metabolism, an ACSL1 shRNA inference vector was constructed. ShACSL1-1928 was transfected into preadipocytes, and cells were collected on day 8 after induction of differentiation. The mRNA expression of the *ACSL1* gene was knocked down in the adipocytes (Figure 6b). The results showed that the mRNA expression of the COX2 gene significantly increased when the mRNA expression of the *ACSL1* gene was decreased (Figure 6c). The results of Western blot were similar. The protein expression of ACSL1 decreased, and the protein expression of COX2 significantly increased (Figure 6d).

**Figure 7:**
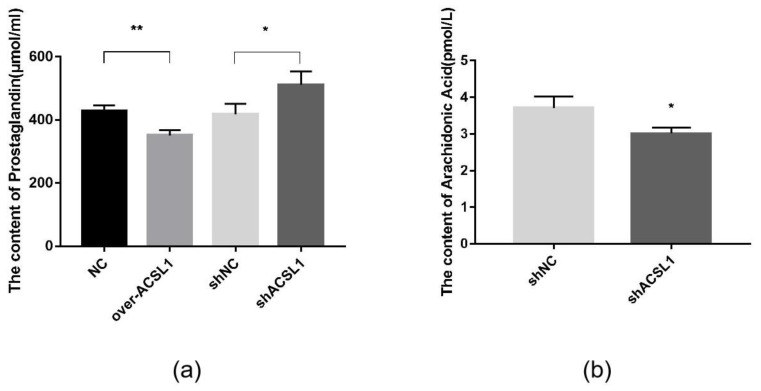
(**a**) The detection of intracellular prostaglandin content (* *p* < 0.05, ** *p* < 0.01); (**b**) The detection of intracellular AA content (* *p* < 0.05). shNC: Adipocytes transfected with shNC as control. shACSL1: Adipocytes transfected with shRNA-1928 of the *ACSL1* gene.

Prostaglandins are downstream metabolites of AA, which is regulated by *COX2*. The AA and prostaglandin content were detected using an ELISA kit. Prostaglandin content in the overexpression group was slightly lower than the control group (Figure 7a). When *ACSL1* was knocked down, AA content decreased, and prostaglandin content increased compared to the controls (Figure 7a,b).

**Figure 8:**
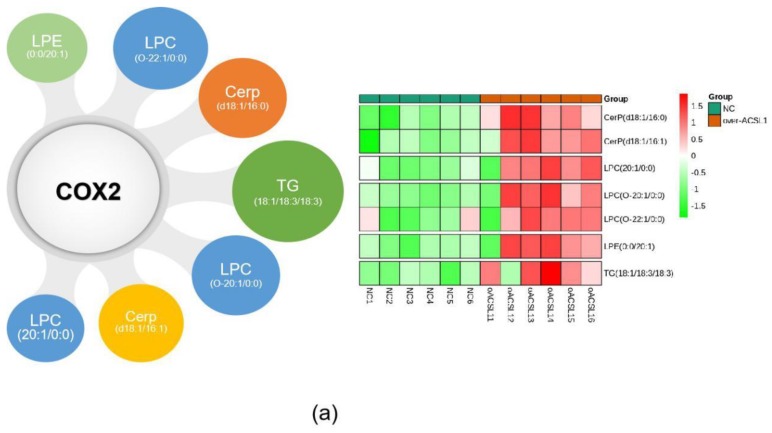

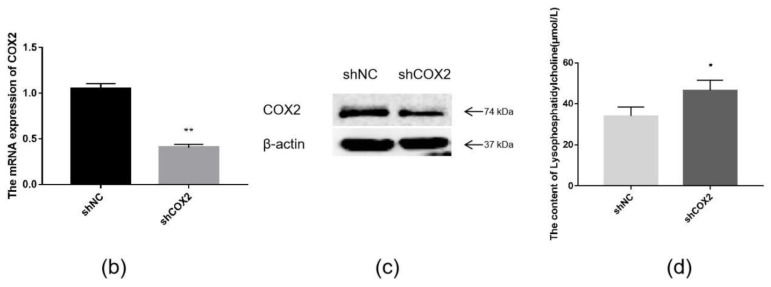
(**a**) Joint analysis of differential metabolites associated with *COX2*; (**b**) *COX2* mRNA expression on day 8 of differentiation (** *p* < 0.01); (**c**) COX2 protein expression on day 8 of differentiation; (**d**) the detection of intracellular LPC content (* *p* < 0.05). shNC: Adipocytes transfected with shNC as control. ShCOX2: Adipocytes transfected with shRNA-1676 of the *COX2* gene.

The combination of transcriptome and lipid metabolome analyses indicated that the differential metabolites associated with the *COX2* gene were CerP (d18:1/16:0), LPC (20:1/0:0), CerP (d18:1/16:1), TG (18:0/18:2/18:3), LPC (O-20:1/0:0), LPC (O-22:1/0:0), and LPE (0:0/20:1) (Figure 8a). These findings suggest that changes in these metabolites may be caused by the overexpression of ACSL1, and there are some correlations between the expression of *COX2* gene and these lipid metabolites. ShCOX2-1676 was transfected into preadipocytes, and cells were collected on day 8 after induction of differentiation. The mRNA expression of the *COX2* gene was knocked down in the adipocytes (Figure 8b). The results of Western blot were similar (Figure 8c). When *COX2* was knocked down, LPC content increased compared to the controls (Figure 8d).

### 2.4. Expression of Genes after Exogenous Addition of AA

**Figure 9:**
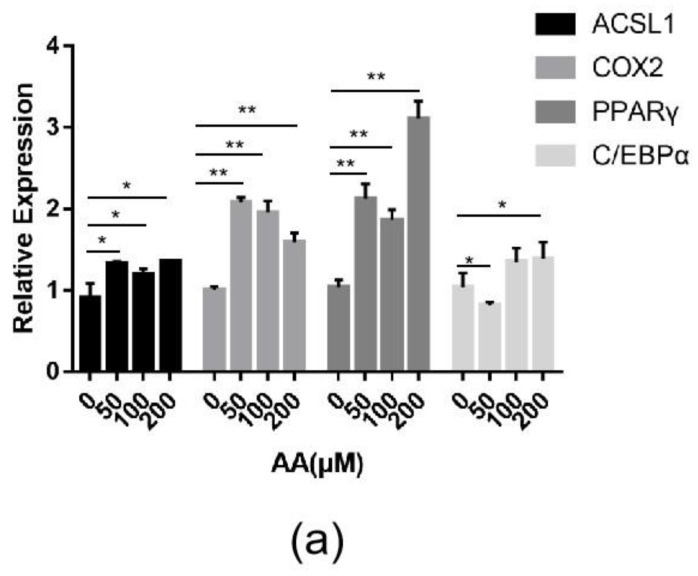

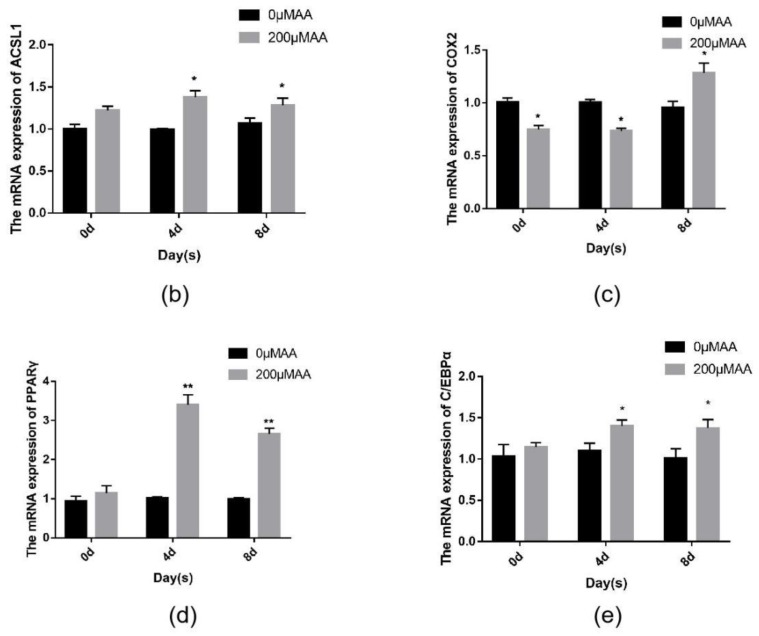
(**a**) *ACSL1*, *COX2*, *PPARγ*, and *C/EBPα* mRNA expression levels in sheep adipocytes with different concentrations (50, 100, and 200 μΜ) of AA on the 9th day of differentiation; (**b**–**e**) *ACSL1*, *COX2*, *PPARγ* and *C/EBPα* mRNA expression levels in sheep adipocytes during different stages of differentiation (* *p* < 0.05, ** *p* < 0.01).

Changes in the expression of the *ACSL1* and *COX2* genes and the effect of AA on sheep adipocytes were determined by increasing the content of AA exogenously. Adding different concentrations (50, 100, and 200 μΜ) of exogenous AA had roughly the same effect on adipocyte differentiation (Figure 9a). The expressions of *ACSL1, COX2, PPARγ*, and *C/EBPα* significantly increased in the experimental group. The mRNA expression of *PPARγ* upregulated almost three-fold compared to the controls. After adding AA, the expression levels of *ACSL1, PPARγ*, and *C/EBPα* in differentiating preadipocytes were significantly higher on days 0, 4, and 8 compared to the controls (Figure 9b–d). There was a significant change in *PPARγ* in the experimental group compared to the control group on day 4 of differentiation (Figure 9d). The expression level of *COX2* in the AA group was significantly lower than the control group during the early stage of differentiation, whereas *COX2* expression was higher than the control group after differentiation (Figure 9c).

## 3. Discussion

Many lipid metabolism-related genes have been verified to be related to mutton quality, which is important for promoting the mutton sheep breeding industry. Molecular breeding has been widely used in the sheep industry, such as multi-fetal sheep breeding. The polymorphism of *FecB, BMPR-IB*, and *BMP-15*, which is a multiple breeding marker gene, plays an important role in multiple breeding [16,17]. Similarly, sheep can be used as a model to study disease. For example, sheep have been used as a model to demonstrate that maternal malnutrition can induce fetal lipid metabolism disorder and inhibit fetal metabolic development [18].

In the current study, the transcript of the *ACSL1* gene in sheep adipocytes was obtained by rapid amplification of cDNA ends. The transcript used in this study contains a 3’UTR of 1570 bp. It has been previously shown that genes with longer 3’UTRs are more stable and less susceptible to activation [19,20], which may explain why the effect of this transcript on lipid metabolism-related genes in sheep adipocytes was not particularly significant. The 3’UTR may bind to microRNAs such as miR-205, which affects the expression of the *ACSL1* gene [21]. In this study, overexpression of the *ACSL1* gene caused changes in the expression of many genes that are involved in inflammation and immune-related metabolic pathways. Many inflammatory factors are also associated with muscle growth. Interleukin/chemokine receptors, Toll-like receptor members, and tumor necrosis factor receptor family members are all associated with Akt-mediated muscle growth [22]. RNA-seq showed that overexpression of ACSL1 causes some changes in genes involved in the immune pathway, suggesting that the *ACSL1* gene may play an important role in some immune diseases such as lipid metabolism-related diseases and thus requires further investigation. Wang et al. found that the expression level of ACSL1 is significantly associated with the levels of various acyl-CoAs including C16:0-, C18:0-, C18:1-, and C18:2-CoA, triglycerides, and lipids in cancer cells and is elevated in prostate tumors [23]. They also found that the *ACSL1* gene is highly expressed in breast cancer tissues and that the oncoprotein, HBXIP, can upregulate ACSL1 via the transcription factor, SP1 [24]. Aspirin inhibits abnormal lipid metabolism in HCC cells by disrupting NFκB-ACSL1 signaling [25]. These studies suggest that the *ACSL1* gene may affect disease through lipid metabolism.

Metabolome profiling can detect all of the endogenous small molecules involved in metabolism and in maintaining the normal function and growth of the organism, especially small molecular substances with a molecular mass under 1000 kD. The lipid metabolome is a branch of the metabolome, which is an important part of animal and plant metabolism. The main role of the *ACSL1* gene is to activate FAs to promote the synthesis of FA-CoA, while ester acyl-CoA is involved in the synthesis of diglycerides, triglycerides, glycerophospholipids, and beta oxidation of FAs. In the current study, the lipid metabolome profiling showed that FFA(20:3) is an important substrate for ACSL1 was activated and the diglyceride content was significantly upregulated. The results also showed the TG(18:1/18:3/18:3) and TG(18:0/18:2/18:3) triglyceride contents increased, whereas the TG(14:0/14:1/18:1) triglyceride content decreased. Thus, the total amount of triglycerides did not change. Similarly, the current study found that up-regulation of glycerophospholipids suggesting that the *ACSL1* gene may play an important role in the synthesis of glycerophospholipids. Triglyceride synthesis in fat and liver tissues is mainly via the diglyceride pathway [26]. Diglyceride is also an important intermediate product of glycerophospholipids [27]. Triglycerides and glycerophospholipids can be synthesized by the diglyceride pathway, which indicates that the FA-CoA produced by the transcript of the *ACSL1* gene is involved in the diglyceride synthesis pathway. Hepatocytes and adipocytes usually synthesize triglycerides via the diglyceride pathway [28]. Li et al. showed that overexpression of the ACSL1 gene can activate FA and transport FA to diglyceride and phospholipid synthesis but away from cholesterol ester synthesis [29]. ACSL1 promotes the transport of n-3 and n-6 FA in glycerophospholipids [30]. These studies indicate that the *ACSL1* gene plays an important role in the activation of FFA and the synthesis of triglycerides, diglycerides, and glycerophospholipids.

In the lipid metabolome, the content of AA increased in the ACSL1 overexpression group. RNA-seq showed that the mRNA expression of *COX2* was significantly lower in the overexpression group than the control group. It has been shown that AA inhibits the differentiation of preadipocytes via increases in COX1 and COX2 [31]. However, the relationship among ACSL1, COX2, and AA is not clear. Many studies have shown that the *COX2* gene is involved in the differentiation of adipocytes. Both COX subtypes are involved in the negative regulation of adipocyte differentiation [32]. The products of COX, PGE2, and PGF2 inhibit adipocyte differentiation. In the presence of indomethacin, a COX inhibitor, intracellular TG content was found to be significantly higher than in controls [31]. Tumor necrosis factor has also been found to upregulate the expression of COX2 in differentiating 3T3-L1 adipocytes. Inhibition of COX2 activity restores TNF-inhibited differentiation, suggesting that COX2 plays an important role in adipocyte differentiation that can mediate TNF signaling [33]. However, some studies have shown that 3T3-L1 cells cannot differentiate normally after the addition of COX2 inhibitors, and COX2 may play an important role in the early stage of cell differentiation [34]. When *COX2* was knocked down, intracellular LPC content increased compared to the controls. The down-regulation of the COX2 gene may have a role in the process of up-regulation of LPC. Studies have shown that LPC is regulated by COX2 expression in vascular endothelial cells [35]. Moreover, ceramide-induced increase in COX2 transcription is mediated through higher NF-κB activation [36]. Therefore, COX2 gene plays an important role in lipid metabolism.

AA plays a major role in regulating lipid metabolism-related genes [37]. AA is widely distributed in the animal kingdom, a small amount in glycerides and also in glycerophospholipids [18]. In previous studies, *ACSL4*, which is a member of the ACSL family, was thought to be involved in AA metabolism [38,39]. In the current study, the expression of the cell fat marker gene PPARγ significantly increased after the addition of exogenous AA, suggesting that exogenous AA may promote lipogenesis. Dervishi found that changes in FAs during the feeding process affected changes in mRNAs of the lipid metabolism-related genes, which affected the intramuscular FA content [40]. The content of spirulina in the feed can alter intramuscular fat accumulation in sheep. At the same time, it changed the expression levels of BTG2, ADRB3, and FASN in adipose tissues [41]. According to the above research, AA promotes the formation of intermuscular fat, and thus AA may be added to the feed.

The content of AA increased, and the expression of COX2 was significantly lower in the ACSL1 overexpression group. The current study also observed a decrease in prostaglandin content, which is the AA downstream metabolite, indicating that the COX2 metabolic pathway was inhibited and affected the metabolism of AA. This indicates that the increase in AA content is due to the accumulation of blocked metabolites caused by ACSL1 and COX2. At the same time, ACSL1 gene interference vector was constructed to verify the results of overexpression of *ACSL1* gene. All results prove that the increase in AA content is because the overexpression of *ACSL1* gene reduces the expression of COX2 and the AA metabolic pathway is blocked. According to the above results, the main role of the *ACSL1* gene is to activate FAs to promote the synthesis of acyl-CoA, while acyl-CoA is involved in the synthesis of diglycerides, triglycerides, and glycerophospholipids, and ACSL1 regulates the content of AA in adipocytes through COX2. It is not clear how the acly-CoA generated by ACSL1 enters the specific pathway, which is worth further study.

## 4. Materials and Methods

### 4.1. Cell Culture and Sheep Preadipocyte Differentiation

Sheep preadipocytes were separated from the adipose tissues of a 40-day-old sheep. Adipose tissues were collected from the alar of the sheep. The tissues were washed and dissociated using 4% collagenase II (Sigma, St. Louis, MO, USA). All cells were maintained at 37 °C with 5% CO_2_ in DMEM supplemented with 10% FBS and 1% penicillin-streptomycin.

The sheep preadipocytes were induced using the classic cocktail method. When the cells reached confluency (Day 0), these were induced with 10 μg/mL insulin (Sigma), 0.5 mM IBMX (Sigma), and 1 mM Dex (Sigma) for 48 h. The medium was then replaced with inducing medium II, a complete medium with 10 μg/mL insulin. After 48 h, the medium was changed to complete medium and then replaced every 48 h until the cells fully differentiated into mature adipocytes.

### 4.2. Construction of ACSL1 Gene Vector

ORF Finder was used to find the ORF region of a known sequence, and then these were submitted to NEBcutter V2.0 for analysis. Primers were designed for the restriction sites *BamH*I and *EcoR*I using the following sequences: F: 5’-GAGGATCCAGCCATGATGCAAGCCCACGAGCTGTT-3’, R: 5’-GTAGAATTCTTAGACTTTGATGGTGGAGTAAAGC-3’ at the two ends of the ORF region, and the corresponding sequences were added for primer synthesis. T4 ligase was used to ligate the target fragment to the pcDNA3.1(+) vector and sequence.

The ShRNA vector of shACSL1-1928: GGAAGGAGTCTGGCCTGAAAC and shCOX2-1676: GCTGTCCCTTTACCTCATTCA was synthesized by a biological company and ligated to the pGPU6 vector and sequence.

### 4.3. Cell Transfection and Sample Collection

The overexpression vector (1 μg) of ACSL1 was transfected into 1 × 10^6^ preadipocytes by Fugene (Promega, WI, USA). The ratio of vector to transfection reagent is 1:3. Stand for 10 min after mixing, add to the cells gently, and change the medium after 24 h. When the cells had fully differentiated into mature adipocytes on day 8, these were immediately washed with PBS. One milliliter of TRIzol (Thermo Scientific, Waltham, MA, USA) was added to six-well plates and pipetted repeatedly. The cells were then transferred to a 1.5-mL RNase-free centrifuge tube and flash-frozen in liquid nitrogen for subsequent RNA-seq.

At least 10^7^ cells were used in lipid metabolome analysis. The cells were trypsinized or scraped off the plate and then transferred into an EP tube. The supernatant was aspirated out, and the collected cells were washed with pre-cooled PBS and stored at −80 °C.

ShRNA vector (1 μg) of ACSL1 and shRNA vector (1 μg) of COX2 was transfected into 1 × 10^6^ preadipocytes by Fugene. Transfection method is the same as the overexpression vector. The cells were collected when these had fully differentiated into mature adipocytes on day 8.

### 4.4. Sequencing and Data Analysis

A total amount of 3 µg RNA per sample was used as input material for the RNA-seq sample preparations. In the CBOT cluster generation system, the truseq PE cluster toolkit v3-cbot-hs is used to cluster the index coding samples. After the cluster generation, the library preparation is sequenced on the Illumina hiseq platform to generate 125 bp/150 bp paired end reading.

Extraction of intracellular lipids for Metabonomic sequencing. The sample extracts were analyzed using a liquid chromatography-mass spectrometry (LC-MS) system [42].

All data analysis was based on the self-built database MWDB (Metware Biotechnology Co., Ltd. Wuhan, China).

Differential gene expression analysis of two groups (two biological replicates per condition) was performed using the DESeq R package (1.18.0). Genes with an adjusted *p*-value < 0.05 found by DESeq were assigned as differentially expressed.

The results of hierarchical cluster analysis (HCA) of the samples and metabolites were presented as heat maps with dendrograms, whereas Pearson correlation coefficients (PCC) between samples were calculated using the cor function in R and presented as heat maps. Both HCA and PCC were conducted using R package pheatmap. For HCA, normalized signal intensities of metabolites (unit variance scaling) are visualized as a color spectrum.

LC-MS analyses were performed as previously described. Metabolites with a variable important in projection (VIP) ≥1 and fold change ≥1.4 or ≤−1.4 were considered statistically significantly different. VIP values were extracted from the OPLS-DA results, which also contain score plots and permutation plots, and generated using the R package MetaboAnalystR.

Identified genes and metabolites were annotated using KEGG Compound database (http://www.kegg.jp/kegg/compound/); annotated genes and metabolites were then mapped to the KEGG Pathway database (http://www.kegg.jp/kegg/pathway.html). Pathways with significantly regulated genes and metabolites were then fed into metabolite set enrichment analysis (MSEA), and their significance was determined by the hypergeometric test’s *p*-values.

For joint analysis, we selected DEGs and differential metabolites enriched in metabolic pathways with Pearson coefficients (PCCs) ≥0.8.

### 4.5. Detection of Triglyceride Content

The differentiated adipocytes were collected on day 8, and 100 μL of the cell lysate per 1 × 10^6^ cells were added. Intracellular triglyceride content was determined using the triglyceride kit (Beyotime, Beijing, China), and the OD value was measured at a wavelength of 450 nm. The standard curve was used to calculate intracellular triglyceride content.

### 4.6. Oil Red O Staining

The mature adipocytes were washed three times with PBS, fixed in 4% paraformaldehyde and stained with 60% Oil red O solution (Oil red O: isopropanol 3:2, Sigma).

### 4.7. Quantitative Real-Time PCR Analysis

Total RNA of the overexpression and control groups was extracted using TRIzol and reverse transcribed into cDNA (Takara, Tokyo, Japan). Quantitative real-time PCR (qRT-PCR) amplification was performed with SYBR Premix Ex Taq using a Roche 480 LightCycler for analysis of gene expression. The expression of genetic mRNA was analyzed using the 2^−ΔΔCt^ method and normalized to β-actin. Primers are shown in Table 2.

### 4.8. Western Blotting

The cells were collected using protein lysis buffer (RIPA, Thermo Scientific), and total protein concentrations were determined using an Enhanced BCA Protein Assay Kit (Beyotime, Beijing, China). The protein samples were denatured at 95 °C for 10 min, separated in a 12% SDS-PAGE gel and transferred to a polyvinylidene difluoride (PVDF) membrane for 90 min at 200 mA. The membranes were washed thrice with TBST and then blocked using 5% skimmed milk at room temperature for 2 h. The samples were incubated with a primary antibody (ACSL1, Bioss, 1:1,000; COX2, Abcam, 1:500) overnight at 4 °C and with the corresponding secondary antibody (β-actin, CST, 1:2,000) for 1.5 h at room temperature. Membranes were washed thrice with TBST and luminescence treated using the ECL-Plus Kit (Beyotime).

### 4.9. ELISA

An ELISA kit (mlbio, Shanghai, China) was used to determine the total PGs, AA, LPC, and diglyceride content. The kit assesses sheep PG (AA, LPC, Diglyceride) levels in the sample using purified sheep PG (AA, LPC, Diglyceride) antibodies to coat the microtiter plate wells to generate solid-phase antibodies. Then, PG (AA, LPC, Diglyceride) was added to the wells, and the combined PG (AA, LPC, Diglyceride) antibody was HRP-labeled to form an antibody-antigen-enzyme-antibody complex. After washing completely, a TMB substrate solution was added. The TMB substrate became blue in color via HRP enzyme catalysis, and the reaction was terminated by the addition of a sulphuric acid solution; the color change was measured spectrophotometrically at a wavelength of 450 nm. The concentration of sheep PG (AA, LPC, Diglyceride) in the samples was then determined by comparing the OD of the samples to the standard curve. All data are reported as the mean by comparing the OD of the sample to the standard curve. Differences with *p* < 0.05 were considered to be statistically significant.

### 4.10. Differentiation of Sheep Preadipocytes with Arachidonic Acid

Different concentrations of AA (Abcam) (50, 100, and 200 μM) were added before the cells were induced. AA was added with complete medium during the entire induction process, DMSO was added as control, and the cells were collected when fully differentiated into mature adipocytes.

Approximately 200 μM AA were added 48 h before the cells were induced; the cells were then collected on days 0, 4, and 8 of induction.

### 4.11. Statistical Analyses

Three independent biological experiments were conducted and measured, and the corresponding means were calculated. All data are reported as the mean ± SEM, and all statistical analyses were performed using the Student’s *t* test. Differences with a value of *p* < 0.05 were considered statistically significant.

## 5. Conclusions

In this study, triglyceride content did not increase after overexpression of the ACSL1 gene. However, the content of diglyceride, which is involved in the overexpression of ACSL1-induced synthesis of fatty acyl-CoA, significantly increased. Changes in the ACSL1 gene affected COX2 gene expression, thereby influencing AA metabolism.

## Figures and Tables

**Table 1:** The differential metabolites in adipocytes overexpressing the ACSL1 gene compared with the controls.

Compounds	Class	Fold_Change	Type	cpd_ID
FFA(20:5)	Eicosanoid	1.403	up	C06428
FFA(20:4)	Eicosanoid	1.415	up	C00219
FFA(20:3)	FFA	1.429	up	C00162
LPC(20:1/0:0)	LPC	1.623	up	C04230
LPC(20:4/0:0)	LPC	1.412	up	C04230
Tetradecenoyl-carnitine	CAR	1.584	up	--
CerP(d18:1/16:0)	CerP	1.405	up	--
CerP(d18:1/16:1)	CerP	1.407	up	--
DG(14:0/16:0/0:0)	DG	1.446	up	C00641
DG(18:1/18:2/0:0)	DG	1.418	up	C00641
DG(18:1/20:5/0:0)	DG	1.529	up	C00641
LPC(22:2/0:0)	LPC	1.671	up	C04230
LPC(20:5/0:0)	LPC	1.481	up	C04230
LPC(O-20:1/0:0)	LPC-O	1.493	up	--
LPC(O-22:1/0:0)	LPC-O	1.520	up	--
LPE(0:0/20:1)	LPE	1.490	up	C04438
PS(18:0/20:4)	PS	1.421	up	C02737
TG(14:0/14:1/18:1)	TG	0.711	down	C00422
TG(18:0/18:2/18:3)	TG	1.447	up	C00422
TG(18:1/18:3/18:3)	TG	1.459	up	C00422

FFA: free fatty acid; LPC: lysophosphatidylcholine; CAR: acylcarnitine; Cerp: Ceramide; DG: diglyceride; LPE: lysophosphatidylethanolamine; PS: phosphatidylserine; TG: triglyceride.

**Table 2:** Primers for quantitative real-time polymerase chain reaction.

Gene	Sequence/(5′-3′)	Product Size/bp
ACSL1	GCCATCACCTACATCATCAACAA ACACTTCTTGCCTCGTTCCA	151
COX2	AAATGCTGGTGTGGAAGGT TTGTTGCTCTAGGCTTTGCT	107
PPARγ	AAGTCCTTCCCGCTGAC CTCTTTGCTGGGCTCCT	159
C/EBPα	CGTGGAGACGCAACAGAAG AAGATGCCCCGCAGTGT	105
β-actin	GTCCACCTTCCAGCAGAT GCTAACAGTCCGCCTAGAA	96
